# Visualization of murine lymph vessels using photoacoustic imaging with contrast agents

**DOI:** 10.1016/j.pacs.2018.01.001

**Published:** 2018-01-31

**Authors:** Ryo Nagaoka, Takuya Tabata, Shin Yoshizawa, Shin-ichiro Umemura, Yoshifumi Saijo

**Affiliations:** a Biomedical Imaging Laboratory, Graduate School of Biomedical Engineering, Tohoku University, 6-6-05 Aramaki Aza Aoba, Aobaku, Sendai 980-8579, Japan; b Ultrasound Enhanced Nanomedicine Laboratory, Graduate School of Biomedical Engineering, Tohoku University, 6-6-05 Aramaki Aza Aoba, Aobaku, Sendai 980-8579, Japan

**Keywords:** Photoacoustic tomography, Contrast agents, Lymph vessels, 3D-real-time imaging systems, Ultrasonic transducer arrays, Light wavelength dependence of photoacoustic signals

## Abstract

Metastasis frequently occurs even in the early stage of breast cancer. This research studied the feasibility of using photoacoustic (PA) imaging for identifying metastasis in the lymph vessels of mice. The photoacoustic efficiency of various contrast agents was investigated, and the influence of scattered light was evaluated by using a lymph vessel phantom. The lymph vessels of mice were then visualized using the selected contrast agents: indocyanine green (ICG) and gold nanorods (AuNR). The attenuation of the PA imaging was −1.90 dB/mm, whereas that of the fluorescence imaging was −4.45 dB/mm. The results indicate the potential of identifying sentinel lymph nodes by using PA imaging with these contrast agents.

## 1 Introduction

Breast cancer is the most common cancer in women worldwide, accounting for 25% of all cancers in women [1]. Computed tomography (CT), magnetic resonance imaging (MRI), mammography, and ultrasonography are used in breast cancer screening, but early-stage breast cancers less than 10 mm in diameter are difficult to detect. Furthermore, metastasis to distant organs via blood or lymph cannot be ignored and needs to be diagnosed in the early stage. One diagnostic method is to identify the sentinel lymph node, which is the first lymph node to which cancer cells spread from the primary tumor. Besides biopsy [[2], [3]], fluorescence imaging [[4], [5], [6]] and radioactive colloid tracers [7] are used for identifying this node. Fluorescence imaging uses indocyanine green (ICG), which is a cyanine dye. The dye flows through lymph vessels, which have a typical diameter of 100–150 μm, and it allows them to be visualized in real time. The tracer method involves injecting radioactive colloid into the area of the tumor. These methods are not without their problems. Fluorescent light is scattered in tissues, and this degrades the quality of images and accuracy of identification. Radioactive colloid tracers expose patients to radiation.

For these reasons, other means have been sought for visualizing tissues. In particular, photoacoustic (PA) imaging, based on a photo-thermal phenomenon [8], visualizes living tissues selectively by using light at specific wavelengths [9], and it has been the basis of a number of developments. In particular, photoacoustic tomography (PAT) [[10], [11], [12], [13], [14], [15], [16], [17], [18], [19], [20], [21], [22], [23]] has attracted attention for its potential clinical applications and as a means of imaging tissues of small animals. Hybrid optoacoustic tomography [10] has been used for imaging small animals and detecting joint inflammation. A handheld PA imaging system has been used to visualize the vasculatures of small animals [[11], [12], [13], [14], [15]], breast cancer, and human skin [[16], [17], [18], [19], [20]]. While PA imaging systems have been used to visualize tumors and related blood vessels [[21], [22], [23]], they have yet to be used for imaging lymph nodes and lymph, which have not been reported to have an absorption peak at a specific wavelength [[24], [25]]. On the other hand, lymph vessels have been visualized using optical-resolution PA microscopy (OR-PAM) [26], and sentinel lymph nodes have been visualized using acoustic-resolution PAM (AR-PAM) [[27], [28]] with a contrast agent of Evans blue (EB).

Our aim is to identify sentinel lymph nodes in the early stage of breast cancer by visualizing the path from the tumor to the node with a contrast agent (e.g. gold nanorods (AuNR) [[29], [30]], ICG [[31], [32], [33]], or EB [[26], [27], [28]]). In this paper, we describe our studies on visualizing lymph vessels. First, the suitability of various contrast agents for PA imaging was examined. Here, a single focused ultrasound sensor was used to evaluate the efficiency of the contrast agents. Second, the effect of scattered light on PA imaging was evaluated using a lymph vessel phantom. An array transducer was used to visualize volume images of the targets. Finally, the lymph vessels of mice were visualized using a PA imaging system we developed and the chosen contrast agents.

## 2 Materials and methods

### 2.1 Evaluation of candidate contrast agents for PA imaging

PA intensities of six candidate contrast agents, AuNR, ICG, IR780, chlorin e6 (C6), protoporphyrin IX (PPIX), and acridine orange (AO), were measured and compared. The first two agents are widely used for PA imaging: AuNR (amine-terminated, 10 nm diameter, concentration: 1.8 kg/m^3^ (9.138 mM), peak absorption: 808 nm, dispersion in H2O, Sigma-Aldrich Co. LLC.) [[29], [30]] and ICG (081104215, concentration: 2500 kg/m^3^ (3225 mM), peak absorption: 780 nm, hepatic function test, Daiichi Sankyo Company, Limited) [[31], [32], [33]]. Like the first two, the IR780 agent (425311, concentration: 1000 kg/m^3^ (1500 mM), peak absorption: 795 nm, Sigma-Aldrich Co. LLC.) has an absorption peak in the NIR range [34] and high affinity to tumors. The other three are used in photodynamic therapy. They have high affinity to tumors, and their absorption peaks are in the visible range: Chlorin e6 (MFCD08669566, concentration: 1000 kg/m^3^ (1675 mM), peak absorption: 404 nm, photodynamic therapy for tumors in liver and brain, Frontier Scientific Inc.) [35], PPIX (P8293, concentration: 1000 kg/m^3^ (1777 mM), peak absorption: 405 nm, photodynamic therapy for tumors in liver, skin and brain, Sigma-Aldrich Co. LLC.) [36], and AO (A386, concentration: 100 kg/m^3^ (330 mM), peak absorption: 502 nm, photodynamic therapy for bone sarcoma, Dojindo Molecular Technologies, Inc.) [37]. Table 1 summarizes the chemical and optical properties of the contrast agents. The molecular weight of AuNR was left blank because the molar mass of a nanoparticle is difficult to define.

**Table 1:** Chemical and Optical Properties of Contrast Agents.

	contrast agents					
	AuNR^29,30^	ICG^31–33^	IR780^34^	C6^35^	PPIX^36^	AO^37^
molecular weight [g/mol]		775	667	597	563	302
molecular formula	Au	C_43_H_47_N_2_NaO_6_S_2_	C_36_H_44_ClIN_2_	C_34_H_36_N_4_O_6_	C_34_H_34_N_4_O_4_	C_17_H_19_N_3_
peak absorption wavelength [nm]	808	780	795	404	405	502

**Fig. 1:**
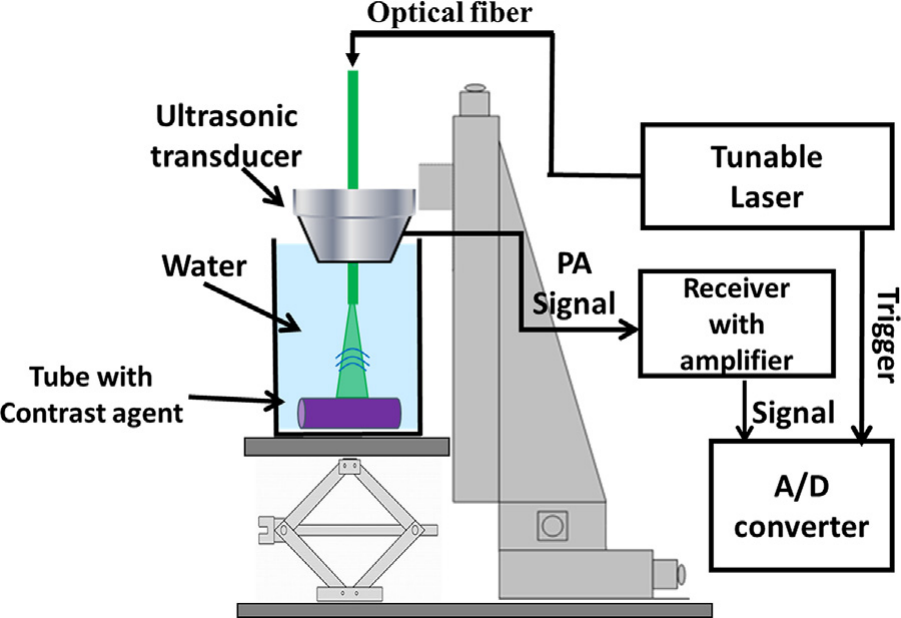
Schematic of experimental setup for photoacoustic measurements of candidate contrast agents.

Fig. 1 shows a schematic diagram of the experimental setup for the acoustic-resolution PA measurements of the contrast agents. A short-pulse (<10 ns) wavelength-tunable optical parametric oscillator (OPO) laser (Opolette 355; 410–2400 nm, 20 Hz, Opotek Inc.) was used for generating the PA signal. The signal was received by a concave poly(vinylidene fluoride/trifluoroethylene) (P(VDF-TrFE)) ultrasound transducer with a central frequency of 50 MHz (−6 dB bandwidth: 10–59 MHz). The aperture size was 4.5 mm, and the focal length was 9.0 mm. The transducer had a hole in its center to insert an optical fiber (effective diameter: 910 μm, wavelength range: 400–2200 nm, 0.22 NA, Photonic Science Technology Inc., Hokkaido, Japan) into and align the illuminating light with the signal detector concentrically. The generated PA signals were amplified 20 dB by a receiver (Honda Electronics Co., Ltd.) and acquired with an A/D converter (DP1400, Acqiris) with a sampling frequency of 1 GHz. The acquisition timing was synchronized with a Q-switched (SW) trigger from the tunable laser. Ten PA signals were averaged for each measurement to improve the S/N ratio. Because ultrasound pulses could be transmitted to the imaging targets, the position of the ultrasonic focus was set to the position of the tube by detecting the maximum-intensity ultrasound signals reflected from the tube before the PA measurement.

The measurement targets were made by injecting 1, 10, 20, 50, 100, and 200-fold diluted contrast agents into a polytetrafluoroethylene (PTFE) tube with inner and outer diameters of 300 and 500 μm. The peak absorption wavelength for each agent was used for each measurement. The imaging target was set at the geometrical focal depth of the ultrasound transducer (9.0 mm). The PA intensity was normalized by the laser output in consideration of the difference in power at different wavelengths. Because of the different concentrations, the efficiency of PA generation was evaluated on the basis of the ratio of the normalized PA amplitude to the concentration. Note that another research group based their evaluation of efficiency on the relationship between the noise equivalent molar concentration and molar extinction coefficient [38].

### 2.2 Measurement of lymph vessel phantom

**Fig. 2:**
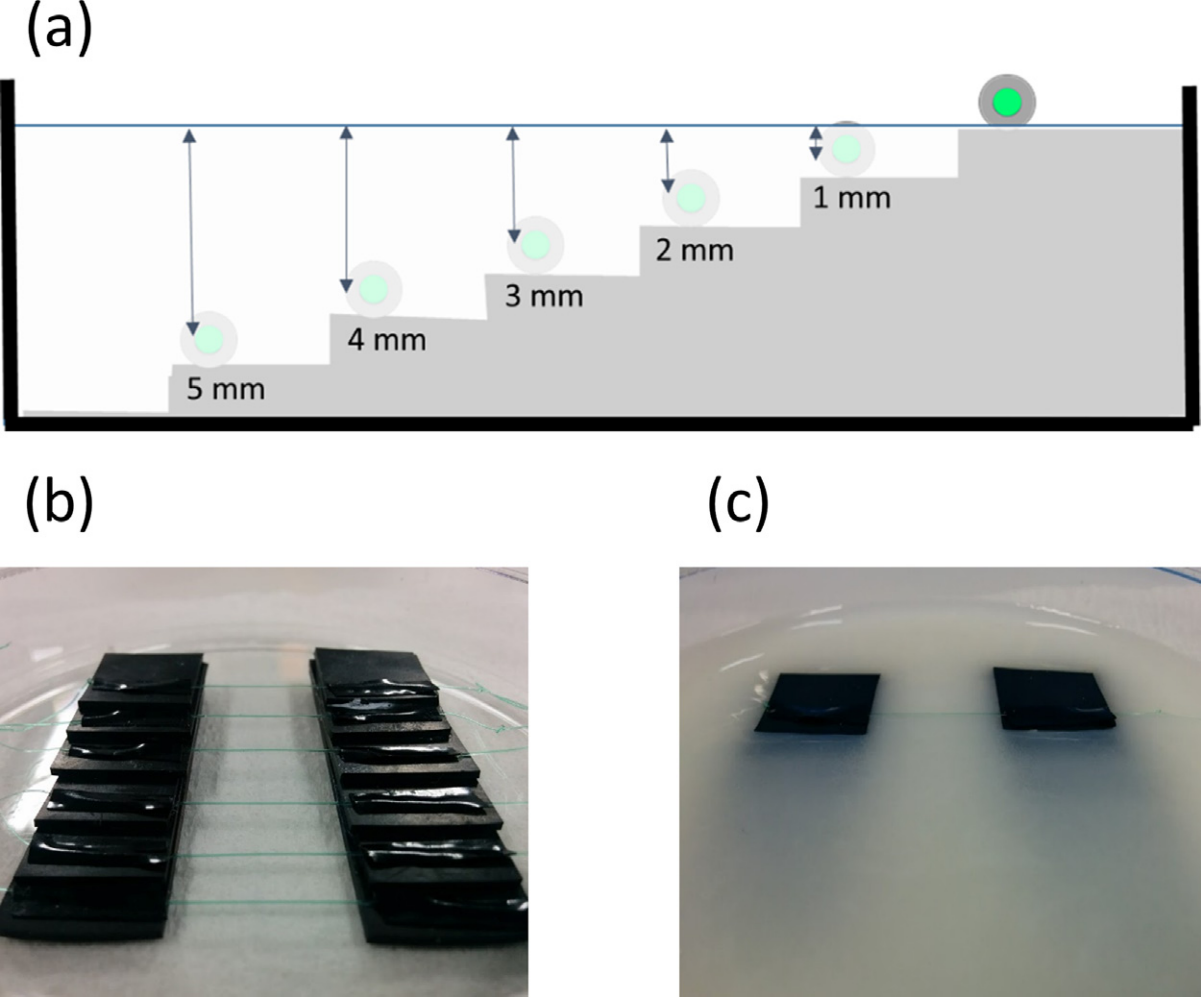
**Lymph vessel phantom.** (a) Schematic diagram of lymph vessel phantom. (b) Configuration of lymph vessel phantom. (c) Lymph vessel phantom filled with 10% intralipid gel.

A lymph vessel phantom (Fig. 2) was fabricated to evaluate the effect of light scattering on PA imaging. The phantom had six PTFE tubes arranged in a staircase pattern at depths of 0, 1, 2, 3, 4, and 5 mm. The tubes had an inner diameter of 100 μm, close to that of lymph vessels, and were injected with ICG (Fig. 2(a, b)). The phantom was filled with a 10% intralipid gel (Fig. 2(c)) [[25], [39], [40]]. The light scattering effect (scattering coefficient μ_s_: 30 mm^−1^ @ 800 nm) in the intralipid gel is similar to that in living tissues.

**Fig. 3:**
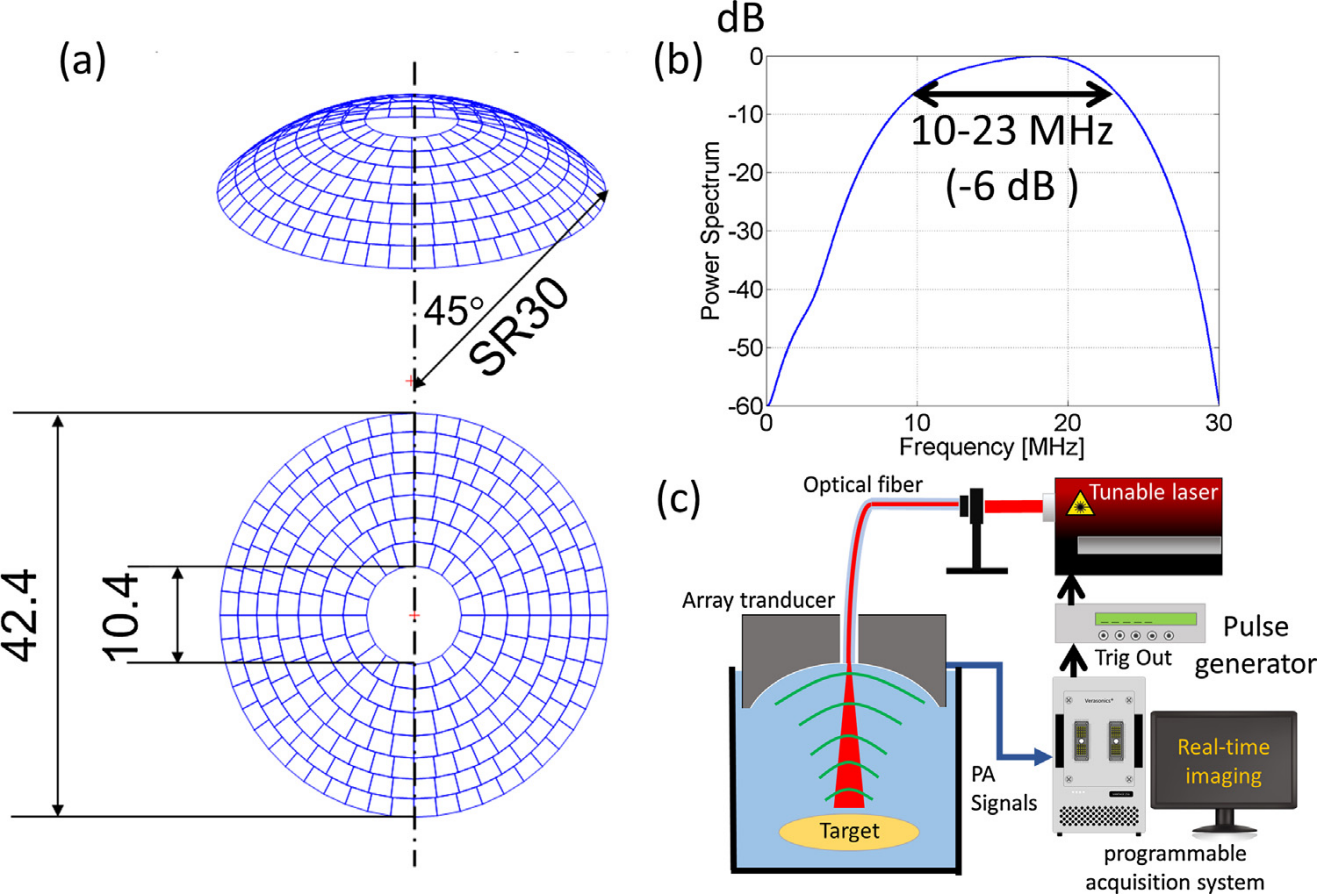
**Real-time PA imaging system.** (a) Geometric arrangement of spherical-curvature array transducer. (b) Impulse response of US detector. (c) Experimental setup of real-time PA imaging system.

Fig. 3 shows the real-time PA imaging system [[19], [20]]. Fig. 3(a) shows the geometric arrangement of the spherical-curvature array transducer consisting of 256 elements made of 1–3-composite material (Japan Probe Co., Ltd.). The transducer geometric had a focal depth of 30 mm and a 10.4-mm hole in the center through which to irradiate targets with a laser. Fig. 3(b) shows an impulse response of the US detector in the frequency domain. The bandwidth was 10–23 MHz (−6 dB range), and the center frequency was 16.5 MHz. Fig. 3(c) shows the experimental setup of the real-time PA imaging system. The short-pulse wavelength-tunable OPO laser was used for generating the PA signals. The PA signals were acquired using a programmable acquisition system (256 Tx/Rx channels, Vantage 256, Verasonics Inc.) with a sampling frequency of 62.5 MHz. The acquisition system was connected to a pulse generator (DG535, Stanford Research Systems Inc.) to synchronize it with the laser irradiation. The synchronization permitted 3D PA imaging in real time at 20 vols per second (vps). The PA images were reconstructed by applying the delay-and-sum (DAS) beamforming method to the acquired signals. In this method, the signals received by each ultrasonic detector array element are summed after compensating for the different travelling times from the elements. The spatial resolution of the system, defined as the full-width at half-maximum (FWHM) of the PA intensities when measuring a single 50-μm-polyamide particle, was 100 × 100 × 100 μm^3^ [19]. The fluence from the optical fiber (effective diameter: 910 μm, wavelength range: 400–2200 nm, 0.22NA, Photonic Science Technology Inc.) was adjusted to satisfy the laser safety standard for clinical measurements [41]. The optical fiber was set at the hole of the transducer. The wavelength was 780 nm, and the repetition frequency was 20 Hz.

The light scattering effects in PA imaging were compared with those in fluorescence imaging (IVIS Spectrum In Vivo System, PerkinElmer Inc.). The fluorescence imaging system had an excitation wavelength of 745 nm, emission wavelength of 820 nm, and exposure time of 1 s.

### 2.3 Visualization of lymph vessels by using PA imaging with contrast agents

The lymph vessels of mice were visualized using the real-time PA imaging system with the chosen contrast agents and with the fluorescence imaging system for comparison. Two Jcl/ICR female mice (2 weeks of age) were used in this experiment. The animals were treated in accordance with guidelines approved by the committee on animal experiments of Tohoku University. All surgical processes were conducted under anesthesia with ketamine (100–120 mg/kg) and xylazine (8–10 mg/kg). The hair was removed from the abdomen and both hind limbs of each mouse with an electric shaver and depilatory cream. The contrast agents were injected into the lymph vessels via a subiliac lymph node (SiLN). The lymph vessels with the contrast agents were visualized from the skin surface.

## 3 Results

### 3.1 Evaluation of candidate contrast agents for PA imaging

**Fig. 4:**
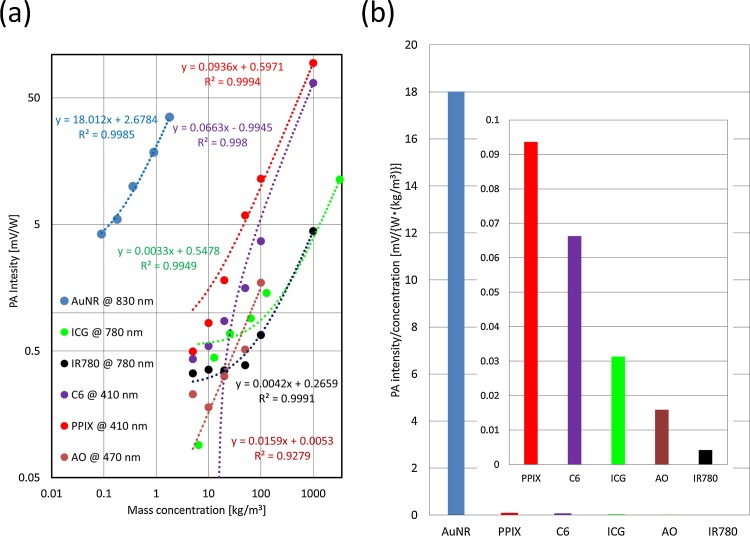
**(a) Relationship among PA intensities from contrast agents, and (b) ratio of normalized PA intensity to concentration.** PA intensity of each contrast agent was generated using laser emitting at peak absorption wavelength. Linear approximate equations were estimated from measured PA intensity. r^2^ is square of the correlation coefficient.

Fig. 4(a) shows the relationship between concentration and PA intensity for each of the six candidate contrast agents. Linear equations and squares of the correlation coefficient R^2^ values were obtained by applying the least-squares method to the measured PA intensities. Nonlinearity in the PA intensity [42] was not detected within the range of concentrations in this experiment. Fig. 4(b) shows the relationship between concentration and the ratio of the PA intensity normalized by both the laser output and concentration. AuNR had considerable PA efficiency. Although PPIX, C6, ICG, AO, and IR780 were all expected to be effective contrast agents, the effects the main light absorbers in living tissues, i.e., water, haemoglobin (Hb), lipid, melanin, and collagen [[24], [25]], are minimized at wavelengths between 700 and 1000 nm. Accordingly, AuNR and ICG were chosen as the contrast agents for PA imaging.

### 3.2 Measurement results of lymph vessel phantom

**Fig. 5:**
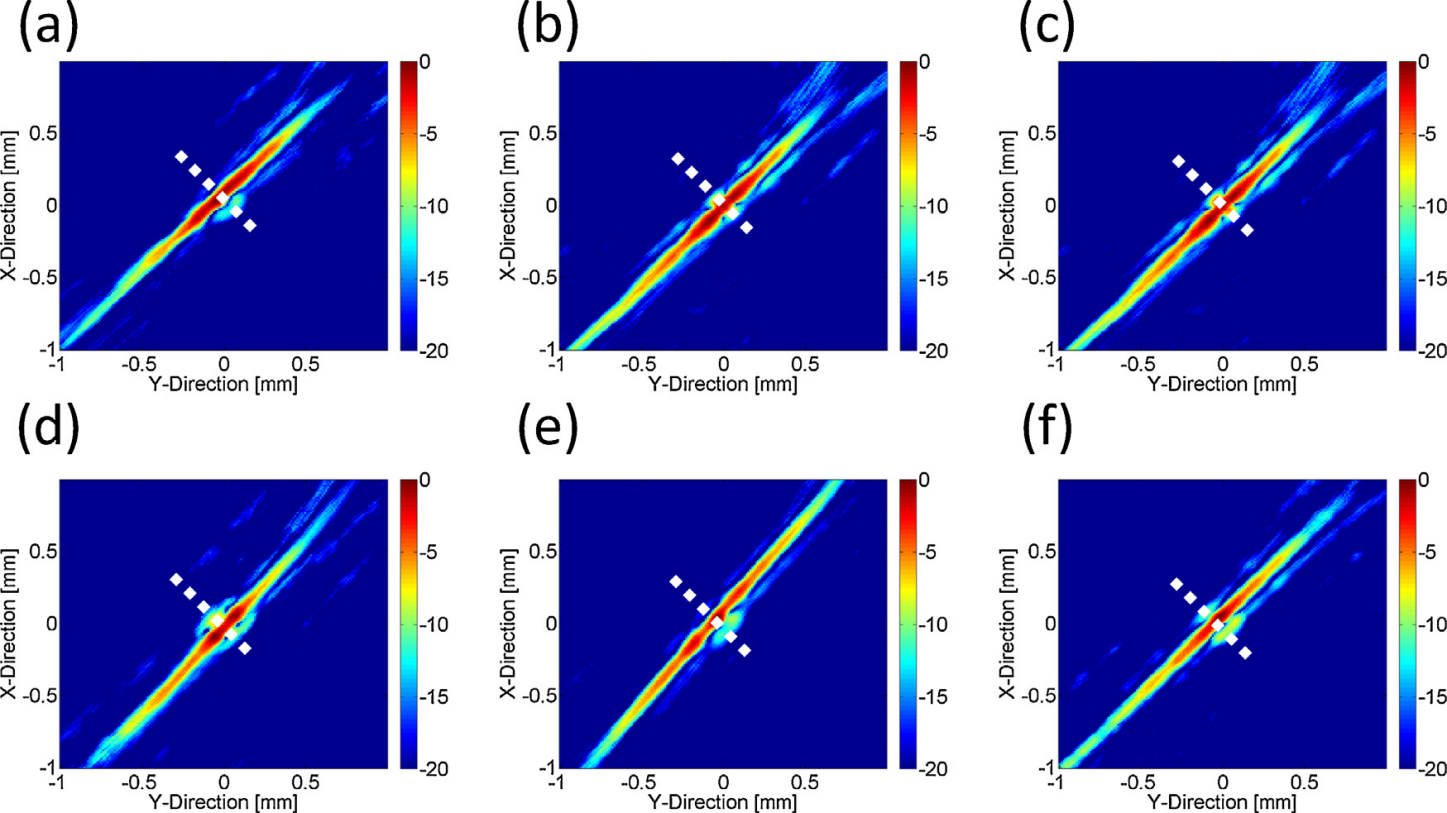
**Measurement results of PA imaging.** PA C-mode images of lymph vessel phantom at depths of (a) 0, (b) 1, (c) 2, (d) 3, (e) 4, and (f) 5 mm.

**Fig. 6:**
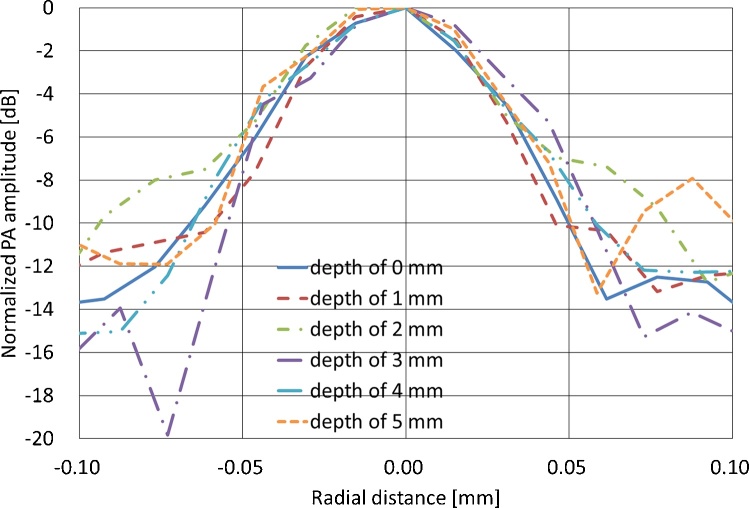
**Comparison of spatial resolution at different depths.** Envelopes of lymph phantom were compared at different depths.

**Fig. 7:**
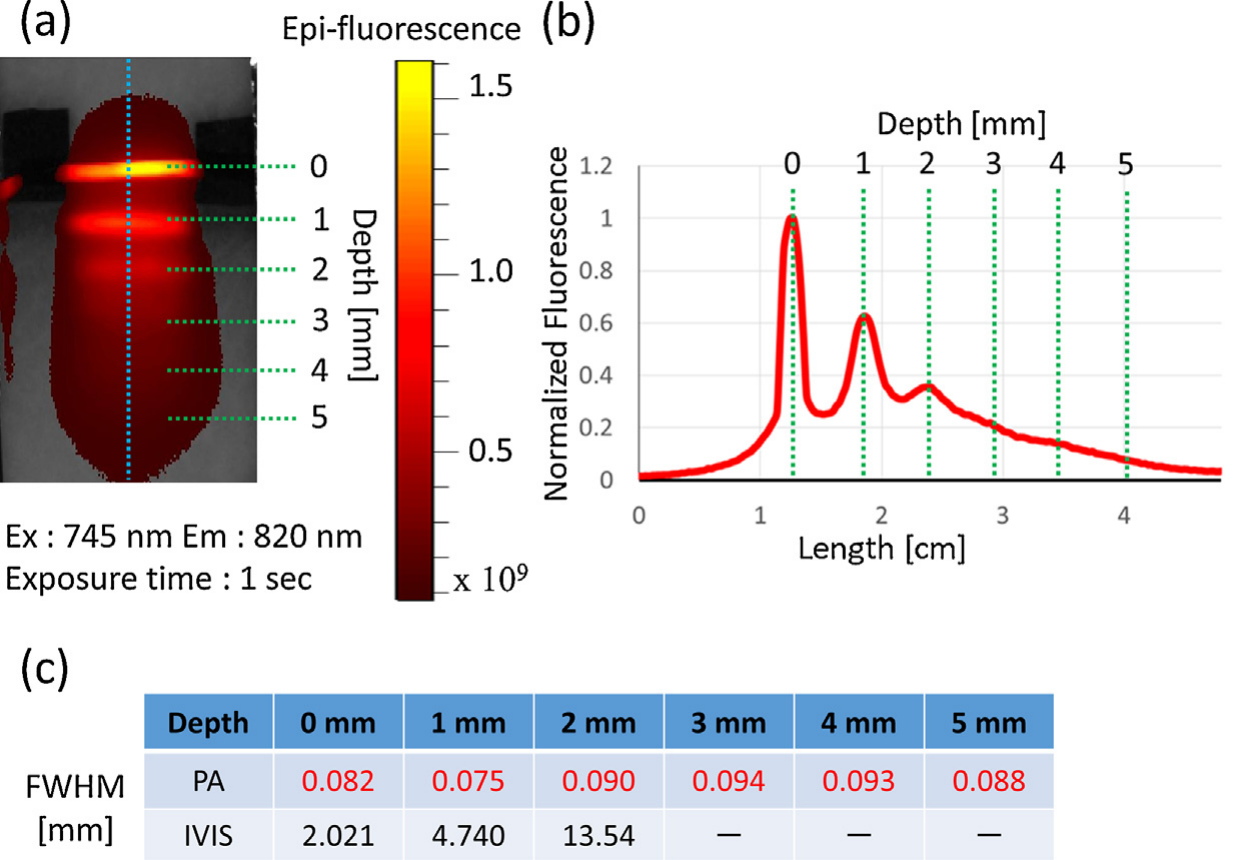
**Results of fluorescence imaging of phantom.** (a) Fluorescence image of lymph vessel phantom. Excitation wavelength was 745 nm, and emission wavelength was 820 nm. Exposure time was 1 s. (b) Normalized fluorescence amplitude along blue dotted line in (a). (c) Comparison of spatial resolutions in PA and fluorescence imaging.

**Fig. 8:**
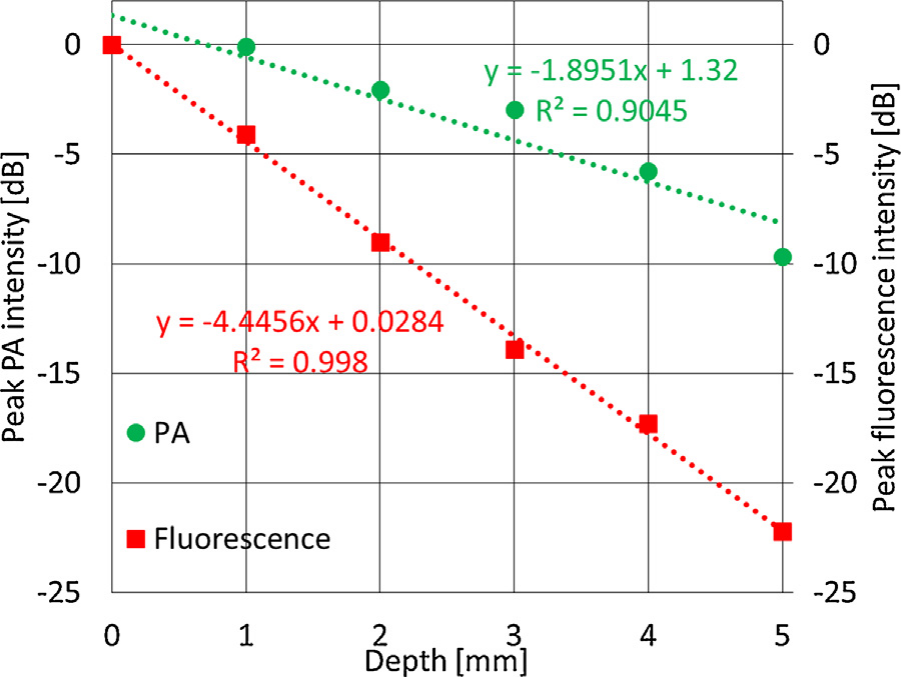
**Comparison of scattering effects between PA and fluorescence imaging.** Linear approximate equations were estimated from measured PA intensity. R^2^ corresponds to power of correlation coefficients.

Fig. 5 shows PA C-mode images of the lymph vessel phantom at depths of 0, 1, 2, 3, 4, and 5 mm. The lymph vessel phantom could be clearly visualized even at a depth of 5 mm. Fig. 6 shows normalized envelopes along the dotted lines in Fig. 5. Fig. 7(a) shows a fluorescence image of the lymph vessel phantom, in which the vessel could be visualized at depths down to 2 mm. Fig. 7(b) shows an envelope along the dotted line in Fig. 7(a). Fig. 7(c) compares the FWHMs calculated from Figs. Fig. 66 and 77(b). The FWHMs can be regarded as measures of spatial resolution. Those of the PA imaging were close to the diameter of the PTFE tubes with injected ICG. On the other hand, those of the fluorescence imaging overestimated the diameter. This is because the light scattered by the tissues affected the quality of the fluorescence imaging. Fig. 8 compares the scattering effects between PA and fluorescence imaging. Linear approximations and R^2^ values were estimated by applying the least-squares method to the measured PA intensities of the lymph vessel phantom. According to the linear approximations, the attenuation coefficient of PA imaging was −1.90 dB/mm, while that of fluorescence imaging was −4.45 dB/mm. Hence, the attenuation due to light scattering affects PA imaging less than it does fluorescence imaging.

### 3.3 Visualization of lymph vessels by PA imaging with ICG/AuNR

**Fig. 9:**
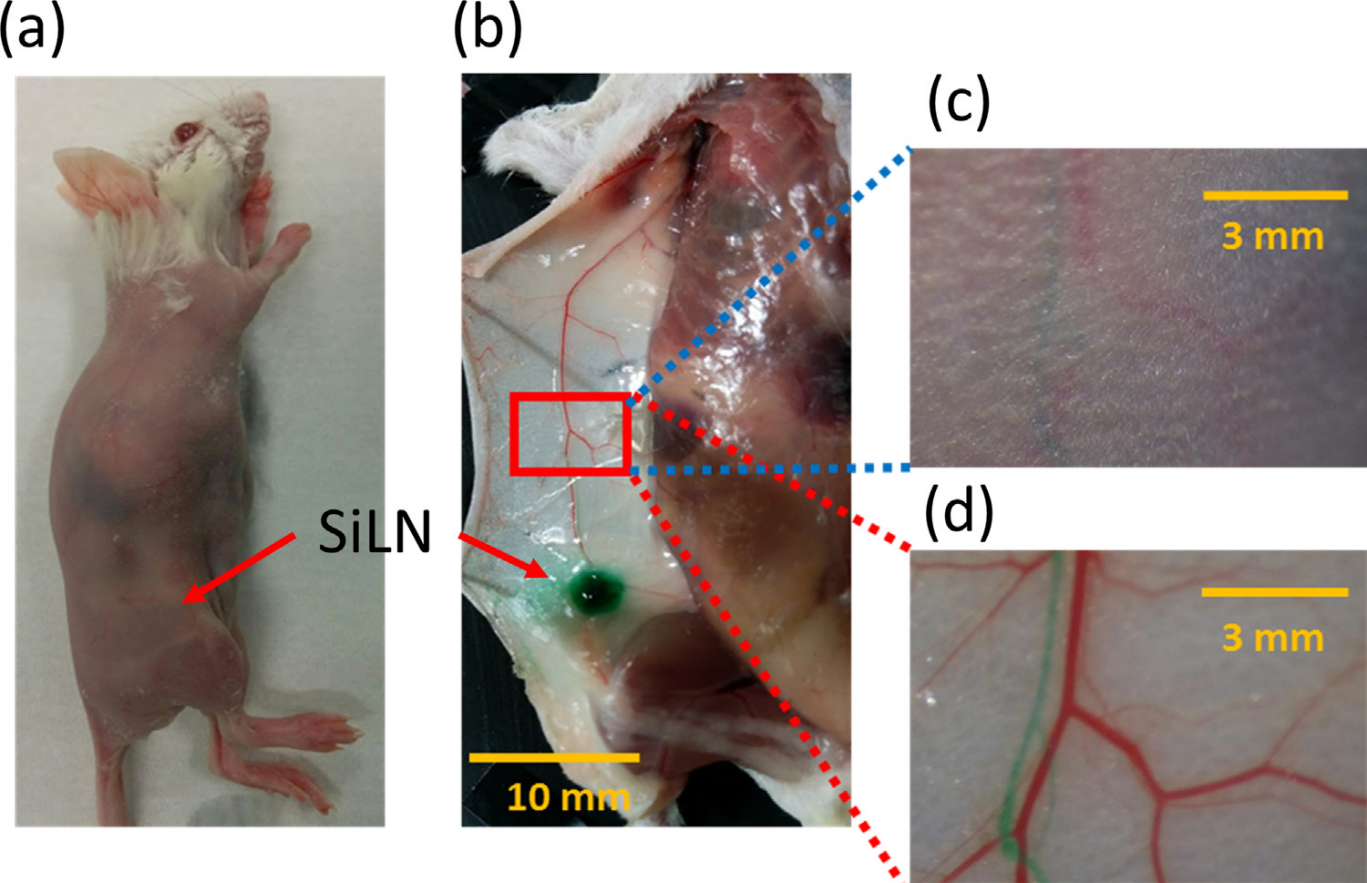
**Optical images of mouse.** (a) Hair-removed Jcl/ICR mouse (2 weeks old). (b) Optical image of lymphatic vessel and superficial epigastric vein. IGC/AuNR were injected into lymphatic vessel via SiLN. For this optical image, ICG was injected into SiLN. (c) Enlarged optical image of skin surface. (d) Enlarged optical image of inner side of skin. Green-dyed vessels are lymphatic vessels.

**Fig. 10:**
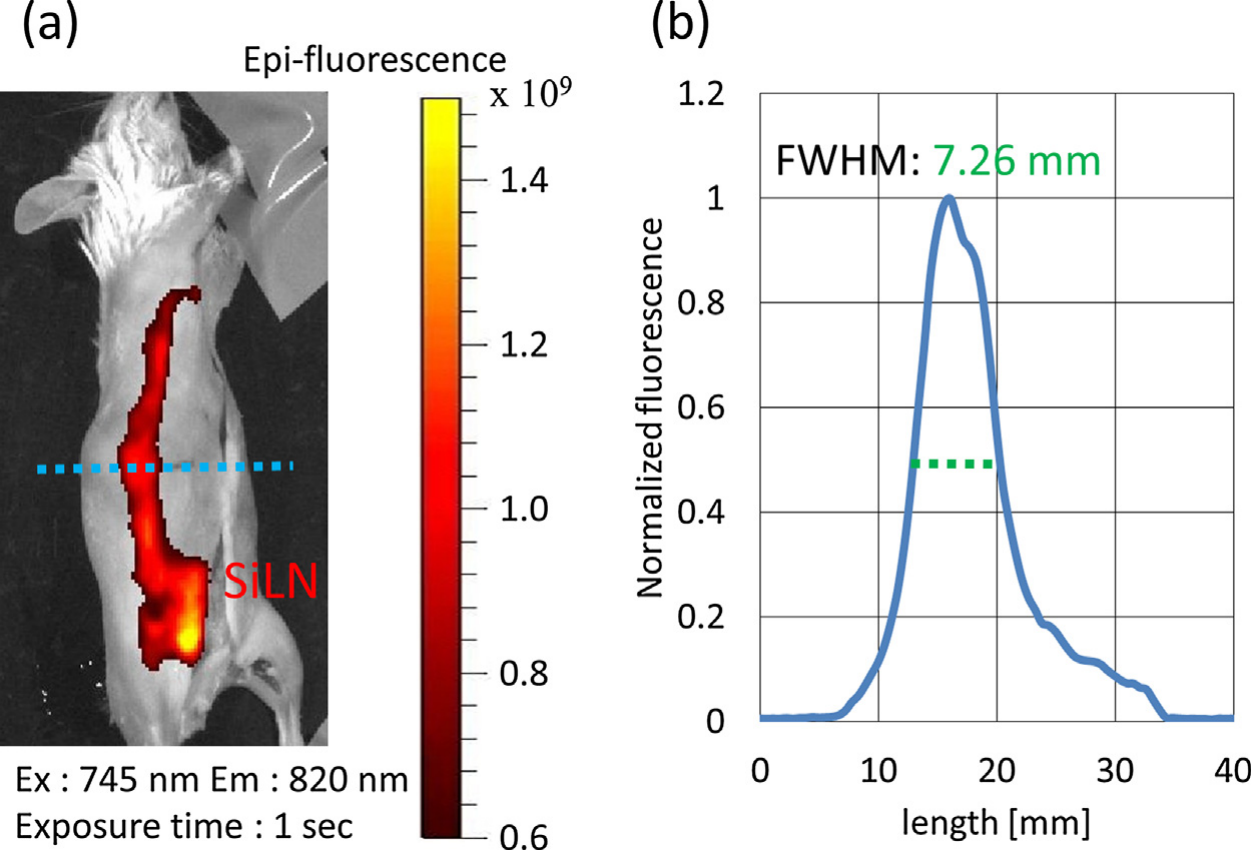
**Fluorescence images of lymph vessel of mouse.** (a) Fluorescent image of lymph vessel of mouse. Excitation wavelength was 745 nm, emission wavelength was 820 nm, and exposure time was 1 s. (b) Normalized fluorescence amplitude along blue dotted line in (a). FWHM was 7.26 mm.

**Fig. 11:**
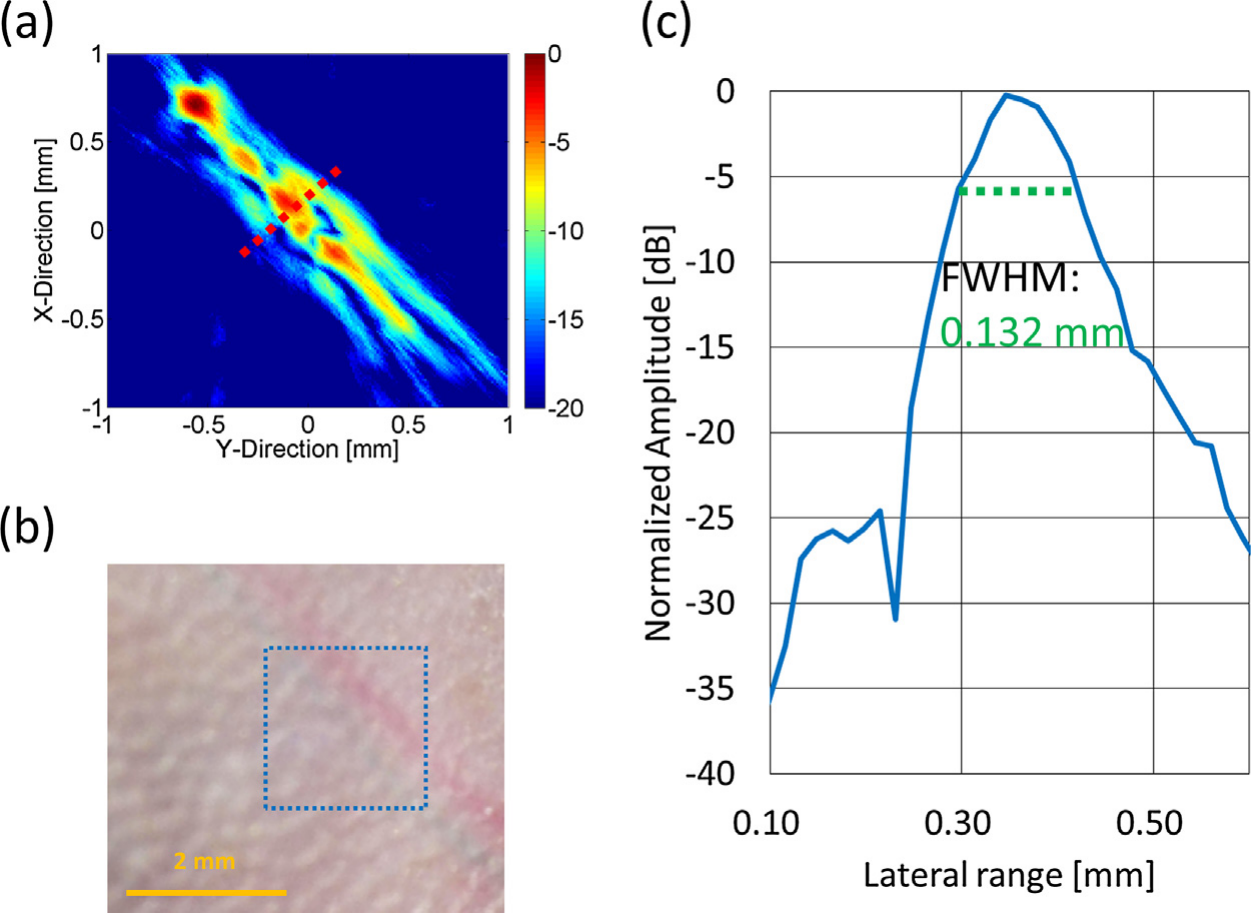
**PA imaging of ICG-injected lymph vessel of mouse.** (a) PA image of ICG-injected lymph vessel of mouse. Laser with wavelength of 830 nm was used for visualizing lymph vessel. (b) Optical image of imaging area. (c) Normalized PA amplitude along red dot line of (a). FWHM was 0.132 mm.

Fig. 9 shows optical images of one of the mice (N = 2). ICG (25 kg/m^3^ (32.25 mM)) was injected into the SiLN, and the dyed tissue is evident in Fig. 9(b). Enlarged optical images of a lymphatic vessel and a superficial epigastric vein as seen from the skin surface and from the inner side of the skin are shown in Figs. Fig. 9(c) and (d). Fig. 10(a) shows a fluorescence image of the ICG-dyed lymph vessel of the mouse. The fluorescence intensity was highest from the SiLN. Fig. 10(b) plots the normalized intensity along the blue dotted line in Fig. 10(a); the FWHM was 7.26 mm. Fig. 11(a) shows the PA image of an ICG-dyed lymph vessel. The laser wavelength was 830 nm. Fig. 11(b) shows an optical image of the imaging area as seen from the skin surface. Fig. 11(c) plots the normalized PA intensity along the red dotted line in Fig. 11(a); the FWHM was 0.132 mm. The diameters of lymph vessels of mice are typically 100–150 μm; the measured FWHMs hence show that the lymph vessel was much more clearly visualized by PA imaging with ICG.

Wavelengths selected at intervals of 20 nm in the range of 510–950 nm were used to visualize the contrast-agent-injected lymph vessel and superficial epigastric vein. The measured PA wavelength-dependence intensities were compared with the absorption spectrum of Hb in previous research [[24], [25]], and the spectra of the contrast agents (ICG/AuNR) were measured using an UV–vis–NIR spectrophotometer (UV-1800, Shimadzu Corp.).

**Fig. 12:**
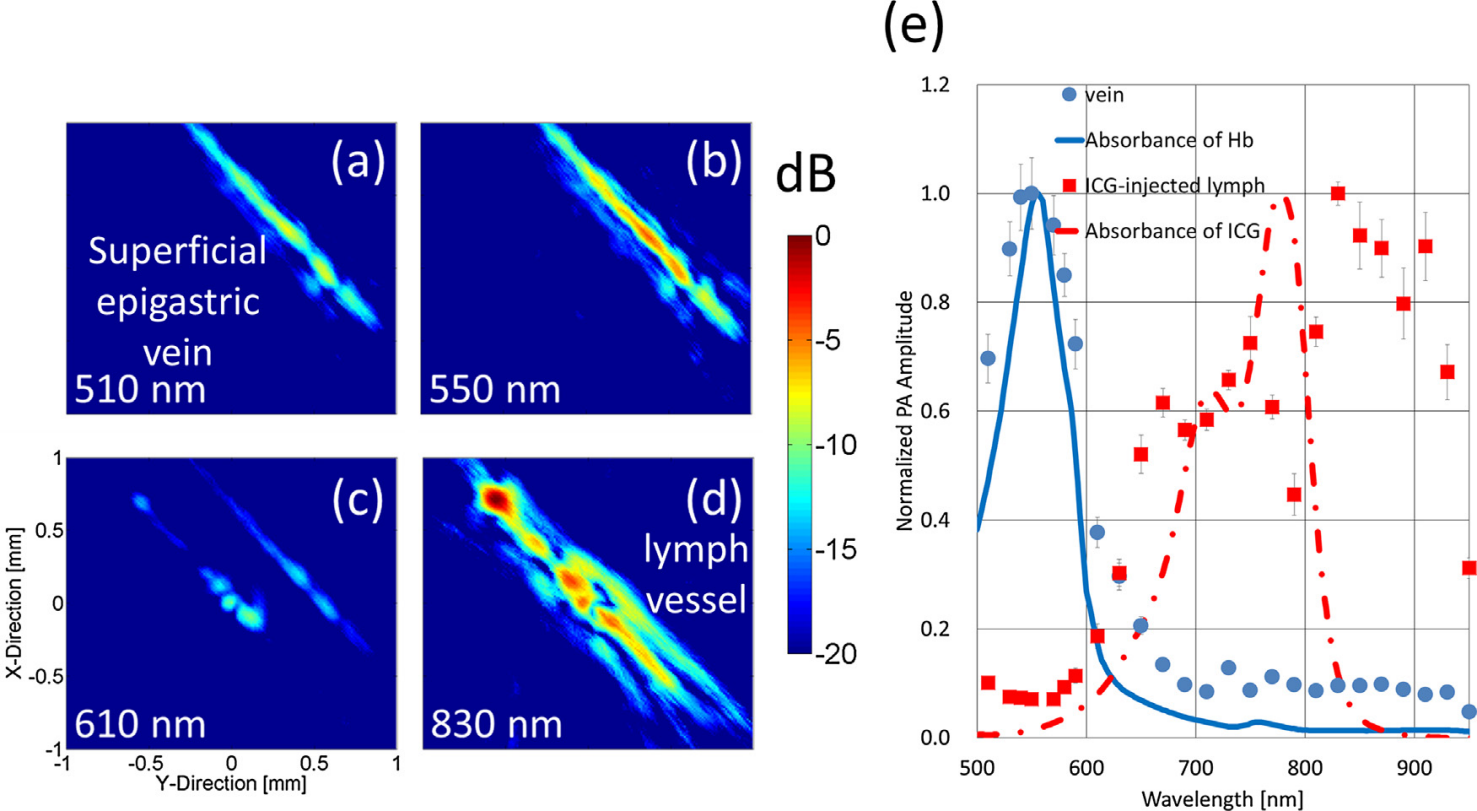
**Visualization results of lymph vessel by PA imaging with ICG.** PA wavelength-dependence C-mode images of ICG-injected lymph vessel and superficial epigastric vein at (a) 510, (b) 550 (peak absorption wavelength of Hb), (c) 610, and (d) 830 nm (peak absorption wavelength of ICG). (e) Comparison results between absorption spectrum by using spectrophotometer (lines) and PA intensity at different wavelengths (dots).

Fig. 12 shows the results of the lymph vessel visualization using PA imaging with ICG (25 kg/m^3^ (32.25 mM)). Fig. 12(a)–(d) show C-mode images made using wavelengths of 510, 550, 610, and 830 nm, respectively. The superficial epigastric vein could be visualized using the wavelength of 550 nm, which almost corresponded to the peak absorption wavelength of Hb in Fig. 12(b). The ICG-injected lymph vessel could be visualized using the wavelength of 830 nm, which almost corresponded to the peak absorption wavelength of ICG in Fig. 12(d). Neither target could be visualized using the wavelength of 610 nm, because the absorption was too low. Fig. 12(e) compares the absorption spectrum acquired with the spectrophotometer (lines) and PA intensity at each wavelength (dots). Each PA intensity was normalized by the maximum intensity from each imaging target. The blue dots correspond to the PA intensities from the vein, and the red squares correspond to the PA intensities of the ICG-injected lymph vessel. The blue solid line corresponds to the absorbance of Hb, and the red dotted line corresponds to the absorbance of ICG. The measured PA intensities of the vein conformed well to the absorption spectrum of Hb. However, there was not much of insignificant difference between the PA intensity of ICG and absorbance of ICG.

**Fig. 13:**
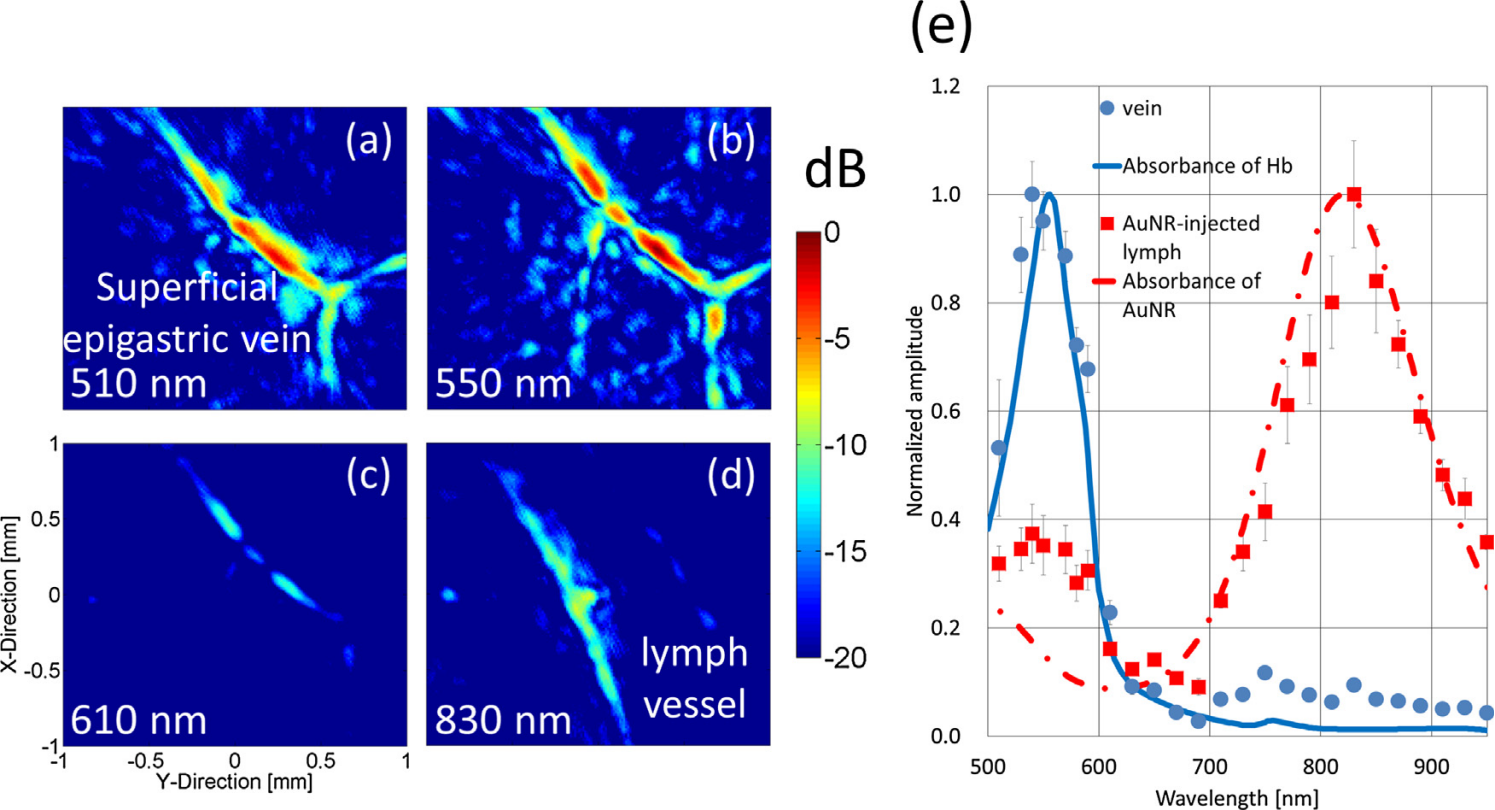
**Visualization results of lymph vessel by PA imaging with AuNR**. PA wavelength-dependence C-mode images of AuNR-injected lymph vessel and superficial epigastric vein at (a) 510, (b) 550 (peak absorption wavelength of the Hb), (c) 610, and (d) 830 nm (first peak with long axis of the AuNR). (e) Comparison results between absorption spectrum by using spectrophotometer (lines) and PA intensity at different wavelengths (dots).

Fig. 13 shows the results of the lymph vessel visualization using PA imaging with AuNR (0.36 kg/m^3^ (1.82 mM)). Fig. 13(a)–(d) show C-mode images made using wavelengths of 510, 550, 610, and 830 nm, respectively. The superficial epigastric vein could be visualized using the wavelength of 550 nm, which almost corresponded to the peak absorption wavelength of Hb in Fig. 13(b), and the AuNR-injected lymph vessel could be visualized using the wavelength of 830 nm, which almost corresponded to the peak absorption wavelength of AuNR in Fig. 13(d). Neither target could be visualized using the wavelength of 610 nm because of the low absorption. Fig. 13(e) compares the absorption spectrum acquired with the spectrophotometer (lines) and PA intensity at different wavelengths (dots). Each PA intensity was normalized by the maximum intensity of the imaging target (vein or AuNR-injected lymph vessel). The blue dots correspond to the PA intensities of the vein, and the red squares correspond to the PA intensities of the AuNR-injected lymph vessel. The blue solid line corresponds to the absorbance of the Hb, and the red dotted line corresponds to the absorbance of the AuNR. Each PA intensity was an average value for a region of interest (ROI) (0.1 × 0.1 mm) that was set at the position of maximum PA intensity in the vein, ICG-injected lymph vessel, or AuNR-injected lymph vessel. Both PA amplitudes conformed well to the absorption spectrum. The PA C-mode image at the wavelength of 830 nm is shown in Figs. Fig. 1212(d) and Fig. 1313(d) because it was the highest in the measurement range.

## 4 Discussion

### 4.1 Efficiency of AuNR

Fig. 4 illustrates that AuNR is very efficient for generating PA signals. Localized surface plasmon resonances result in strong absorption properties and enhance the efficiency of PA signal generation [[43], [44], [45], [46], [47]]. The initial pressure *p*_0_ of the PA signals can be computed as [48]

(1) $$p_{0}=\;\Gamma \eta_{th}\mu_{a}F\text{,}$$

where *Γ* is the Grüneisen parameter, *η*_th_ is the percentage of absorbed light converted into heat, *μ*_a_ is the optical absorption coefficient (cm^−1^), and *F* is the local optical fluence (J/cm^2^). Because the main component of AuNR is gold, the *η*_th_ of AuNR is much higher than that of contrast agents made of organic materials.

A comparison of the maximum PA intensities from the injected lymph vessels normalized by the concentration (ICG: 25 kg/m^3^ (32.25 mM), AuNR: 0.36 kg/m^3^ (1.82 mM)) in the PA wavelength-dependence C-mode images shows that AuNR was almost 380 times more efficient than ICG (ICG: 830 nm, AuNR: 830 nm). The efficiency was close to what was expected from Fig. 4. Hence, the effectiveness of AuNR for PA imaging was confirmed even in an *in vivo* situation.

The ICG agent has been used in clinical practice, mainly for testing hepatic function, and its toxicity is low. However, the concentration of ICG in the mouse experiments was 25 kg/m^3^, whereas clinical dosages are limited to no more than 50 mg. Here, liposomally formulated ICG derivatives [49] and micelles [50] can improve the generation efficiency and decrease the dosage in clinical applications. Moreover, although AuNR has been reported to be toxic, certain coatings reduce its toxicity in bio imaging and photo-thermal therapy [[51], [52], [53]]. Hence, we conclude that ICG and coated AuNR have excellent potential as contrast agents for PA imaging.

### 4.2 Difference between PA intensity and absorbance of ICG

It is considered that plasma in lymph vessels affects the absorption spectra. A previous study [25] examined changes in the absorption spectra for different ICG concentrations in plasma and found that the primary absorption peak shifted to red relative to the water absorption properties.

## 5 Conclusions

The ICG and AuNR agents were identified as suitable contrast agents for PA imaging on the basis of efficiency of their PA signal generation and strong absorption in the proper wavelength region (700–1000 nm). The effect of light scattering on PA and fluorescence imaging was evaluated using a lymph vessel phantom, and light scattering in tissues was found to degrade the quality of fluorescence imaging. The attenuation coefficient of PA imaging was −1.90 dB/mm, whereas that of fluorescence imaging was −4.45 dB/mm. Hence, PA imaging was comparatively affected less by scattering and its attenuation was lower. The lymph vessels of a mouse were visualized using ICG and AuNR and our PA imaging system. The lymph vessels were clearly visualized with ICG. These results indicate that a PA imaging system with ICG and AuNR is potentially useful for identifying sentinel lymph nodes when breast cancer metastasizes.

## Conflicts of interest

The authors declare that there are no conflicts of interest.
